# New directions in mulberry leaf research for diabetes: a translational approach based on multi-component synergy

**DOI:** 10.3389/fnut.2026.1792767

**Published:** 2026-04-13

**Authors:** Jia Chen, Wei Li, Yaqin Jiang, Qichang Xing

**Affiliations:** Department of Pharmacy, The Central Hospital of Xiangtan (The affiliated hospital of Hunan University), Xiangtan, Hunan, China

**Keywords:** 1-DNJ, clinical practice, diabetes, mulberry leaf, multicomponent synergy

## Abstract

The prevalence of diabetes mellitus is increasing year by year globally, but the existing drugs have limitations, and there is still a great demand for natural, complementary hypoglycemic strategies. Mulberry leaves have a long history of use in traditional Chinese medicine for “Xiaoke.” The aim of this review is to summarize the functional constituents with hypoglycemic potential in mulberry leaves, to explore their multiple mechanisms of action, to evaluate the preclinical and clinical evidence, and to discuss the prospects and challenges of their application as dietary supplements. Mulberry leaves are rich in a variety of functional constituents, which show good potential to assist in glycemic control through multi-target pathways such as inhibition of carbohydrate digestive enzymes, regulation of glucose absorption, improvement of insulin sensitivity, protection of pancreatic β-cells and anti-oxidative stress. However, more high-quality clinical studies are needed to standardize the dosage and formulation.

## 1 Introduction

Diabetes mellitus (DM) is a common metabolic disease, the incidence of which is rapidly increasing worldwide and has become one of the major public health problems threatening human health (1). DM is typically characterized by hyperglycemia, and a prolonged hyperglycemic state can lead to a variety of complications, including cardiovascular disease, nephropathy, retinopathy, and neuropathy (2). Currently, the treatment of DM mainly consists of drug therapy and lifestyle intervention. Commonly used hypoglycemic agents include insulin, sulfonylureas, biguanides, α-glucosidase inhibitors and GLP-1 receptor agonists (3). Of these, insulin injections are the key life-sustaining treatment for patients with type 1 DM, while patients with type 2 DM can control their blood glucose levels with oral hypoglycemic agents or combined insulin therapy. In addition, lifestyle modifications such as proper diet, regular exercise and weight management have been shown to play an important role in the management and prevention of DM (4). However, although existing treatments are able to control blood glucose to a certain extent, they are still unable to completely prevent disease progression and complications. Therefore, the development of new therapeutic strategies and drugs to more effectively restore metabolic homeostasis and prevent diabetes and its complications remains an important direction of current medical research.

The World Health Organization points out that traditional medicine is widely used as a source of primary health care worldwide, especially in many low—and middle-income countries (5). In China, since ancient times, traditional Chinese medicine (TCM) practitioners have used mulberry leaves (ML) to treat “Xiaoke” disease (a syndrome that we can now identify as DM), and the “*Compendium of Materia Medica*” (Bencao Gangmu) states that “decocted in juice and served as a tea, can cure Xiaoke.” Modern research shows that the bioactive components in ML, such as alkaloids, flavonoids, polysaccharides, and polyphenolic compounds, are the main material basis for its medicinal effects (6). ML have been applied in various forms, and have been developed as animal feeds (7), food additives (8) and health products (9), in addition to being used as Chinese herbs for health care and treatment (10).

This review aims to summarize the details of the phytochemicals, molecular mechanisms, as well as safety of ML as a preventive and therapeutic agent or functional food for DM based on the recent research progress all over the world. Furthermore, this review also summarized the research progress on multicomponent synergy and clinical practice of ML for the first time. The possible research directions for further in-depth development and application in glycemic controlling are suggested to enhance the value of ML.

## 2 Main functional components in ML (chemical basis)

The chemical composition of ML is rich and diverse, mainly including alkaloids, flavonoids, phenolic acids, polysaccharides, and a variety of other compounds. They are summarized as follows:

### 2.1 Polyhydroxy alkaloids

Polyhydroxy alkaloids in ML are one of the main active ingredients in antidiabetes. Polyhydroxy alkaloids can be divided into polyhydroxypiperidines, polyhydroxypyrrolidines and polyhydroxynortropanes. The representative component 1-Deoxynojirimycin (1-DNJ) is structurally similar to D-glucose, which can competitively inhibit α-glucosidase activity, delay intestinal carbohydrate absorption, and lower postprandial blood glucose. In addition, 1-DNJ can improve insulin sensitivity and tolerance. A variety of alkaloids have been isolated from ML, such as 1-DNJ, N-methyl-1-DNJ, 2-oxo-α-D-galactopyranoside-1-DNJ, and fagomine. Among them, 1-DNJ and fagomine were more active, with content ranges of 0.401-5.309 mg/g and 0.279–2.300 mg/g, respectively (11).

### 2.2 Flavonoids and their glycosides

Flavonoids are one of the other main active ingredients in ML, and are also the most intensively studied and structurally defined class of compounds in the chemical composition of ML. Flavonoids in ML account for 1%~3% of the dry weight, and the currently isolated flavonoids include astragalin, quercetin, rutin, isoquercitrin, kaempferol, and their related derivatives (12). Among them, rutin and quercetin can improve insulin level, blood glucose level, and pancreatic islet cell function, as well as reduce the incidence of diabetic complications (13, 14).

### 2.3 Phenolic acid

The pharmacologically active phenolic acid compounds in ML include chlorogenic acid, cryptochlorogenic acid, neochlorogenic acid, gallic acid, and caffeic acid, etc (6). The content of total phenolic acids in ML accounts for 80 mg−184.3 mg/100 g of dry leaves, and the content of phenolic acid compounds varies greatly among different varieties, origins, harvesting times, and batches (15). Chlorogenic acid can inhibit insulin resistance and improve insulin sensitivity (16), cryptochlorogenic acid can protect pancreatic islet cells (17), and gallic acid can inhibit α-glucosidase activity, inhibit glucose transporter carriers, and reduce blood glucose concentration (18).

### 2.4 Polysaccharides

ML polysaccharides are another class of active ingredients discovered in recent years. Ren Chunjiu et al. (19) isolated and purified MLPII, a homogeneous polysaccharide fraction with hypoglycemic activity, from ML and analyzed it by high performance liquid chromatography (HPLC), which was mainly composed of five monosaccharides, including mannose, rhamnose, glucose, xylose and arabinose, with a molar ratio of 8.73:1.04:6.53:2.13:1.00. Dai et al. (20) isolated ML polysaccharides (MLP) with a purity of 75% as determined by a standard curve. Chromatographic analysis indicated that MLP is composed of the following monosaccharides, listed in descending order of content: glucose, glucuronic acid, xylose, arabinose, galactose, mannose, galacturonic acid, ribose, fucose, and rhamnose. Furthermore, it was demonstrated that MLP can ameliorate glucose and lipid metabolism disorders through the gut microbiota-bile acid metabolic pathway.

### 2.5 Multicomponent synergy

Recently, more and more scholars began to pay attention to the multicomponent synergistic anti diabetes effect and its mechanism of ML. Zhang Yue et al. (21) extracted 1-DNJ, flavonoids, and polysaccharides with purities of 70.40%, 52.34%, and 32.60%, respectively. These components were combined in a ratio of 1:6:8 to form a mixed-material compound (MMC). MMC administration in a rat model of type 2 diabetes mellitus (T2DM) significantly reduced blood glucose, insulin, and lipid levels. Kang Chae-Won et al. demonstrated in *db/db* mice that ML extract or 1-DNJ supplementation ameliorated muscle insulin resistance and restored skeletal muscle structure, suggesting their potential as alternative therapies for diabetes-related muscular complications (22). Cheng et al. (23) isolated ML total flavonoids with a purity of 58.29% and demonstrated that they can activate brown adipose tissue (BAT) and promote the browning of white adipose tissue (WAT). These processes enhance mitochondrial biogenesis, thermogenesis, fatty acid β-oxidation, and energy expenditure, thereby reducing blood glucose, blood lipids, and body weight, as well as improving insulin resistance, which collectively contribute to anti-DM effects. Although these studies collectively support the anti-diabetic potential of ML, as summarized in Table 1, it is worth noting that the synergistic interactions among its bioactive components have often been inferred rather than rigorously demonstrated. For instance, while the MMC formulation described by Zhang Yue et al. implies component interactions, the original study does not appear to employ established synergy models, such as the Chou-Talalay combination index method or isobologram analysis, to quantitatively distinguish synergistic effects from simple additive effects. Further studies incorporating such pharmacologically validated approaches would help clarify whether true synergy underlies the therapeutic efficacy of ML and its active constituents.

**Table 1:** Anti diabetes experimental evidence of ML extract (multi-component).

No.	Name	Extraction method	Standardized parameters (purity)	Model	Dosage	Hypoglycemic effect	Target or mechanism	Reference
1	Mulberry leaf multi-components (MMC)	Polysaccharides, flavonoids, and 1-DNJ were extracted by water extraction and alcohol precipitation respectively, and then purified using different types of macroporous resins	1-DNJ(70.40%), flavonoids(52.34%), and polysaccharides(32.60%) mixed at a ratio of 1:6:8	T2DM rat	100–200 mg/kg b.w./day	Reduce FBG and HOMA-IR level (Equivalent to metformin)	Regulate the PI-3K/Akt signaling pathway in liver	(21)
2	Mulberry Leaf Extract (MLE)	dried in an oven at 50 °C for 9 h, crushed and then extracted with 20 volumes of distilled water at 90 °C for 4 h	Undefined	db/db mice	200–500 mg/kg b.w./day	Reduce FBG and HOMA-IR level, Improve OGTT (Equivalent to 1-DNJ)	Improve skeletal muscle insulin resistance via the activation of IRS-1/PI3K/Akt Pathway	(22)
3	Mulberry leaf flavonoids (MLFs)	extracted three times with 80% ethanol assisted by ultrasound. Then precipitated by ethanol, AB-8 macroporous resin and dried by rotary evaporation	Flavonoids (58.29%)	db/db mice	300–600 mg/kg b.w./day	Reduce FBG and HOMA-IR level, improve OGTT (Equivalent to metformin)	activate BAT and induce browning of WAT via regulating the AMPK/SIRT1/PGC-1α signaling pathway	(23)
4	Folium Mori extract (FME)	extracted with water and ethanol, followed by ethyl acetate and petroleum ether partitioning, and purified using D101 macroporous resin	130.93 g extract was obtained from 60 kg air dried mulberry leaves, which was rich in flavonoids and polysaccharides	T2DM rat	2 g/kg b.w./day	Reduce FBG and HOMA-IR level, Improve OGTT (Equivalent to rosiglitazone)	Activate the IRS-1/PI3K/Glut-4 signaling pathway in skeletal muscles	(45)
5	Mulberry leaf water extraction (MLWE)	Decocted with 15 times water for 4 h, then concentrated and freeze-dried	1 g extract was extracted and purified from 3 g mulberry leaves, containing total phenol acids 19.02 mg, total flavonoids 24.29 mg, and total polysaccharides 72.59 mg	T2DM mice	666 mg/kg b.w./day	Reduce FBG and HOMA-IR level, improve OGTT (Equivalent to metformin)	regulate gut microbiota dysbiosis	(65)
6	Morus alba leaves ethanol extract (MLE)	Extracted with 95% and 70% alcohol	Containing chlorogenic acid, rutin, isoquercitrin and quercitrin (4,340.0, 307.4, 684.4 and 2,191.8 μg/g in MLE)	T2DM rat	200–1000 mg/kg b.w./day	Reduce FBG and HOMA-IR level, improve OGTT and IPITT (Equivalent to metformin)	protects pancreatic islet cells against dysfunction and death by inducing autophagy	(38)
7	Morus latifolia leaf extract	Extracted with 70% ethanol, then separated with chloroform and ethyl acetate, collect the ethyl acetate layer,	Polyphenols (chlorogenic acid 103.00 mg/g, caffeic acid 4.30 mg/g, coumaric acid 11.61 mg/g, rutin 53.00 mg/g and quercetin 46.19 mg/g)	T2DM rat	250–500 mg/kg b.w./day	Reduce FBG (Slightly weaker than glibenclamide)	unknown	(66)

FBG, fast blood glucose; HOMA-IR, Homeostatic Model Assessment of Insulin Resistance; OGTT, Oral Glucose Tolerance Test; IPITT, Intraperitoneal Insulin Tolerance Test.

## 3 Multiple pharmacological mechanisms underlying the hypoglycemic effect of ML

**Figure 1:**
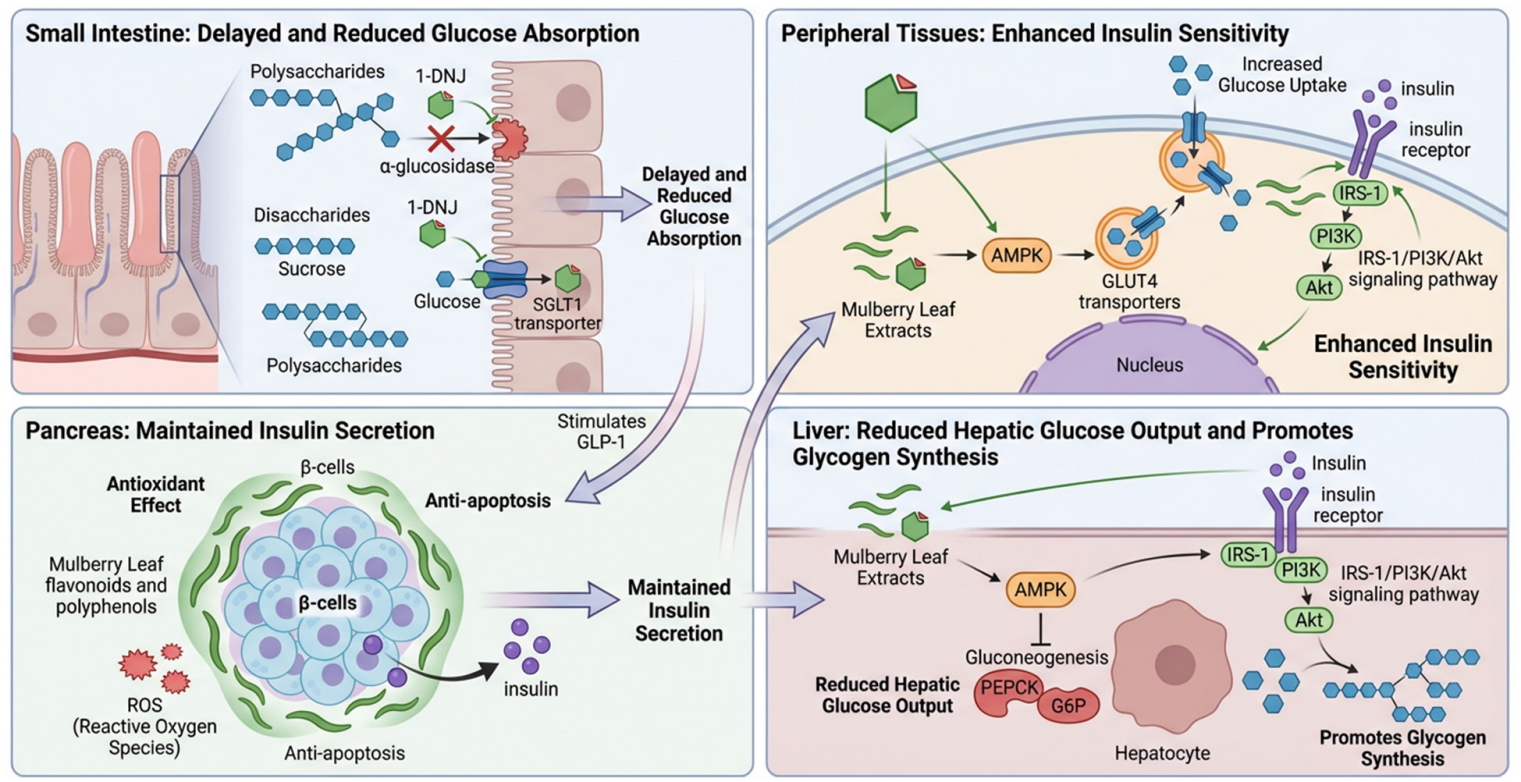
Pharmacological mechanisms of mulberry leaf in glycemic control.

The molecular mechanisms by which mulberry leaves regulate blood glucose are complex and can be summarized into several aspects, as shown in Figure 1.

### 3.1 Inhibits intestinal carbohydrate-digesting enzymes

Inhibition of intestinal carbohydrate-digesting enzymes by ML is the most classic and well-defined mechanism by which it aids in postprandial glycemic control (24). At the heart of this mechanism lies 1-DNJ, a unique alkaloid found in ML, which interferes with the intestinal step of the final digestion of carbohydrates by means of “competitive inhibition.” The 1-DNJ primarily inhibits two types of key digestive enzymes: α-glucosidase and α-amylase (25). The former is located at the brush border of small intestinal epithelial cells and is responsible for hydrolyzing dietary disaccharides (e.g., sucrose, maltose) into absorbable monosaccharides (glucose, fructose). 1-DNJ, because of its structural similarity to glucose, acts as a molecular bait that preferentially enters and binds tightly to the active center of the α-glucosidase, forming a stable enzyme. The latter is mainly secreted by saliva and pancreas, and is responsible for the initial decomposition of starch (polysaccharide) into oligosaccharides such as maltose and other oligosaccharides. ML extracts (especially the flavonoids) also have some inhibitory effect on α-glucosidase, but the effect is usually weaker than 1-DNJ (26, 27).

### 3.2 Regulates intestinal glucose absorption and transport

The pharmacological mechanism by which ML regulates intestinal glucose uptake and transport is its second key line of defense in aiding blood glucose control. We propose that this mechanism primarily operates at the following two levels to directly reduce glucose uptake by intestinal epithelial cells: competitive inhibition of glucose transporters and downregulation of glucose transporter protein expression. 1-DNJ in ML not only inhibits digestive enzymes, but also, as a structural analog of sugar, competes with glucose for the sodium/glucose cotransporter protein 1 (SGLT1), a major transporter protein on the brush border of the intestinal epithelial cells (28). On the other hand, 1-DNJ and phenolics of ML can inhibit the gene expression and protein synthesis of SGLT1 and glucose transporter protein 2 (GLUT2), another transporter protein that assists in glucose diffusion out of the cell, by regulating intracellular signaling pathways (29, 30). This means that in the long run, ML can reduce the number of “transporters” responsible for glucose absorption in the intestinal tract, thus radically reducing the glucose transport capacity of the intestinal tract. Through the dual action of “immediate competitive inhibition” and “long-term expression down-regulation,” ML effectively slows down and reduces the rate and total amount of glucose from the intestinal lumen into the blood circulation.

### 3.3 Enhancing insulin sensitivity in peripheral tissues

The pharmacological mechanism by which ML enhances insulin sensitivity in peripheral tissues is central to its profound role in achieving homeostatic regulation of blood glucose. This mechanism improves the response to insulin in target tissues such as adipose and liver through the following two key pathways, including activatiing adenylate-activated protein kinase (AMPK) and insulin signaling pathway. The active ingredients in ML [e.g., alkaloids (31), flavonoids (23, 32)] can directly or indirectly activate AMPK, an intracellular energy receptor that is activated when there is a lack of energy. Activated AMPK causes translocation of glucose transporter protein 4 (GLUT4) to the cell membrane, thereby directly increasing glucose uptake and utilization of blood glucose by adipocytes independent of insulin signaling, and exerting an “insulin sensitization” effect similar to that of exercise. In the liver, the activation of AMPK pathway can inhibit the expression of key enzymes of gluconeogenesis, reduce the over-synthesis of glucose in the liver, and lower the output of blood glucose from the source (33). On the other hand, the polysaccharides (19) and flavonoids (34) from ML alleviate damage to insulin signaling pathways caused by chronic low-grade inflammation and oxidative stress through their antioxidant and anti-inflammatory effects. This helps protect and enhance the efficiency of the classical insulin receptor substrate-1 (IRS-1) /phosphatidylinositol 3-kinase (PI3K) /protein kinase B (Akt) signaling pathway. When this pathway is smoother, insulin's command to “take up glucose” is more efficiently carried out by the cells, as evidenced by increased insulin sensitivity. ML synergistically “opens the alternate pathway” (activating AMPK) and “repairs the main pathway” (enhancing IRS-1/PI3K/Akt pathway), enhances the insulin sensitivity of skeletal muscle, adipose and other peripheral tissues, thereby significantly improving insulin resistance, which is an important molecular basis for its ability to achieve long-lasting and stable glycemic control.

### 3.4 Protection of pancreatic β-cells and regulation of insulin secretion

The pharmacological mechanism of ML to protect pancreatic β-cells and regulate insulin secretion is the key link in its potential to assist in blood glucose control, which mainly focuses on reducing β-cell damage and optimizing its function. Flavonoids [e.g., rutin (35), quercetin (36)] and polyphenols in ML are potent antioxidants. Quercetin can enhance the antioxidant defense capacity of β-cells, directly scavenging excessive reactive oxygen species, thus alleviating the oxidative stress damage caused by the high glucose environment (37). In addition, the active ingredients of ML can protect β-cells from programmed death and maintain their number and functional integrity by regulating autophagy (38) and inhibiting the mitochondrial apoptotic pathway and endoplasmic reticulum stress (39). The inhibition of α-glucosidase by ML and the delayed digestion and absorption of carbohydrates may shift the location of glucose absorption in the intestine backward and more effectively stimulate the secretion of enteric proinsulin such as glucagon-like peptide-1 (GLP-1) from the distal intestine (40). GLP-1 not only potently promotes insulin secretion in a glucose concentration-dependent manner, but also inhibits glucagon secretion and has a protective effect on β-cells (41). Through the dual strategy of “defense” (protecting β-cells from damage) and “empowerment” (optimizing their secretion), ML helps to maintain and improve the body's own insulin secretion. This not only compensates for the aforementioned peripheral insulin resistance, but also improves the core physiological function of blood glucose regulation from the source.

### 3.5 Regulation of hepatic glucose metabolism

The pharmacological mechanism of ML in regulating hepatic glucose metabolism mainly focuses on the core function of the liver as a “blood glucose stabilizer,” and realizes the effect of reducing endogenous glucose output and increasing glucose storage through the bidirectional regulation of inhibiting gluconeogenesis and promoting glycogen synthesis. The core mechanism of action revolves around the regulation of key signaling pathways. The active ingredients of ML (e.g., 1-DNJ, quercetin) activate AMPK, which phosphorylates and inhibits the activity of CREB-transcriptional coactivator 2 (CRTC2), thereby down-regulating the activity of glucose isomerization rate-limiting enzymes, such as phosphoenolpyruvate carboxykinase (PEPCK) and glucose-6-phosphatase (G6P) and other gluconeogenesis rate-limiting enzymes, inhibiting the hepatic conversion of non-sugar substances into glucose from the source (42–44). In addition, ML improves systemic and hepatic insulin sensitivity through its antioxidant and anti-inflammatory effects, resulting in more efficient IRS-1/PI3K/Akt signaling pathway transduction (45). Activated Akt phosphorylates and inhibits glycogen synthase kinase-3β (GSK-3β), thereby relieving GSK-3β inhibition of glycogen synthase, resulting in enhanced glycogen synthase activity and accelerated conversion of glucose to glycogen for storage (22). There are also some studies suggesting that components such as ML polysaccharides may also directly promote glycogen synthesis through insulin-independent pathways such as PI3K/Akt (19).

## 4 Clinical evidence

The role of ML in aiding glycemic control has been validated in several clinical trials, and the evidence for its efficacy and safety is becoming increasingly clear. Several randomized controlled trials (RCTs) have shown that taking ML extract with or before a meal significantly reduces postprandial blood glucose compared to placebo (46–48). This is largely attributed to its immediate effect of inhibiting intestinal α-glucosidase. Cui et al. (49) conducted a systematic review and meta-analysis of 12 RCTs involving 615 participants to evaluate the effects of ML or ML extract (MLE) on glycemic traits. The meta-analysis demonstrated that supplementation with ML/MLE significantly reduced fasting blood glucose (FBG) by −0.47 mmol·L^−1^, glycosylated hemoglobin (HbA1c) by −2.92 mmol·mol^−1^, and fasting plasma insulin by −0.58 μIU·ml^−1^. Subgroup analysis further indicated that long-term supplementation (≥8 weeks) was particularly effective in non-healthy individuals and those with baseline FBG >6.1 mmol·L^−1^. The hypoglycemic effect of ML showed a trend of “postprandial fasting glycated hemoglobin”, which is in line with its “intestinal-first, multi-target-assisted” mechanism of action. However, it is important to note that among the trials included in this meta-analysis, only one study (46) explicitly reported using an extract standardized to a specific 1-DNJ content, while the remaining studies did not specify the standardization of their ML preparations to bioactive markers. This lack of consistent reporting on extract composition underscores a critical gap between the promising clinical efficacy observed and the challenges in ensuring product quality and reproducibility, which will be discussed further in the context of standardization. More broadly, the current clinical evidence base is limited by several factors, including relatively small sample sizes, short intervention periods, and considerable heterogeneity in the preparation and composition of the ML formulations used across different studies. These methodological limitations highlight the need for larger, longer-term, and better-controlled trials with well-characterized extracts to more definitively establish the efficacy of ML for glycemic management.

In terms of safety, summarizing the available clinical trial data, ML shows good safety and tolerability at the conventional recommended dose (50). ML is classified as a “medicinal and food source” substance by the Chinese Health Commission, and its long history of consumption provides a basis for its safety (51). No serious adverse reactions associated with ML were reported in clinical trials. Common adverse events: mainly gastrointestinal related, including mild bloating, bowel sounds, increased flatulence or loose stools (52), which is due to the fermentation of undigested carbohydrates in the colon by flora and is similar in nature to the adverse effects of α-glucosidase inhibitor drugs such as acarbose. These symptoms are usually mild, transient, and lessen as the body adjusts. However, when used in combination with sulfonylureas or insulin, there may be a superimposed hypoglycemic effect and the risk of hypoglycemia needs to be guarded against. Therefore, diabetic patients should be under the supervision of a physician when combining medications and intensify blood glucose monitoring.

## 5 Forms and challenges of application as an adjunctive glycemic control agent

ML is increasingly used as an adjunctive glycemic control agent, but still faces the core challenge of standardization in its actual promotion.

### 5.1 Main forms of application

ML tea (53, 54) is the most common and acceptable form, which is consumed by brewing, but the rate of dissolution of the active ingredient (e.g., DNJ) and the dosage are not easy to control. Standardized extracts in the form of capsules, tablets or powders, usually labeled with the content of key ingredients such as 1-DNJ, which is the main direction to achieve precise dosage and controllable effect (55, 56). Third, functional food additives which were added to food products such as rice (57), cookies (58), and beverages (59), aiming to achieve auxiliary regulation in daily diet. However, it is worth noting that many of the bioactive compounds in ML, particularly polyphenols and flavonoids, are susceptible to thermal degradation during high-temperature processes such as baking or drying (60, 61). To address this formulation challenge, recent study has explored novel delivery systems, microencapsulation technologies, to protect these heat-sensitive components and enhance their stability during food processing (62). Such approaches not only preserve the bioactivity of ML constituents but also improve their controlled release and bioavailability, thereby supporting the development of more effective functional food products.

### 5.2 Core challenges and limitations

In terms of standardization and quality control, the content of 1-DNJ and other active ingredients in ML is significantly affected by the variety, place of origin, harvesting season, and processing methods (63), which leads to instability in the quality of raw materials and products. Recent studies have demonstrated that drying techniques profoundly influence the retention of heat-sensitive bioactive compounds in ML. Zhao et al. systematically compared five drying methods and found that instrumental drying (oven, freeze, microwave) was superior to natural processes (sun, air) in preserving bioactivity. Notably, freeze, and microwave drying were most effective at retaining total polyphenols, flavonoids, and alkaloids, whereas oven drying excelled in preserving amino acids (64). Regarding 1-DNJ, an important alkaloid with hypoglycemic activity, previous research has shown that its content is highly susceptible to thermal degradation during prolonged drying processes (67). Moreover, there is a lack of recognized quality control standards for hypoglycemic efficacy (e.g., minimum effective 1-DNJ content labeling) for marketed products, making it difficult to compare and guarantee the effects among different products. In addition, the doses used in clinical trials have been shown to be effective, precise dose ranges for different populations (e.g., pre-diabetic and diabetic patients with different stages of diabetes) have not yet been established. Further, ML has a potential risk of hypoglycemia when combined with hypoglycemic agents and needs to be used with caution under monitoring. Therefore, it needs to be clearly emphasized that ML is a complement to lifestyle interventions and pharmacological treatments, not a substitute. If not managed properly, it may lead to overdependence and neglect of standardized treatment.

## 6 Future research directions and prospects

As a natural resource with a long history of application and clear multi-target hypoglycemic potential, future research on ML should deepen from “knowing what it is” to “knowing why it is,” and promote its development toward standardization, precision, industrialization and evidence-based development. On the one hand, we should continue to deepen the basic research: from macro-effects to micro-mechanisms and networks, including the use of systemic pharmacology, metabolomics and network pharmacology methods, analysis of alkaloids, flavonoids, polysaccharides and other multi-component interaction networks in ML, to clarify the precise molecular basis of its “multi-target, low toxicity” synergistic effect. In addition, explore the role of ML in regulating chronic low-grade inflammation (e.g., M1/M2 macrophage polarization) in adipose tissue, liver and other metabolic organs, and elucidate its upstream regulatory mechanism in metabolic inflammation. On the other hand, it is necessary to promote clinical translation by conducting large-scale, long-period, high-quality RCTs, focusing on hard endpoint outcomes such as cardiovascular events and renal protection, and evaluating the benefit/risk ratio of their long-term (≥1 year) application. Systematically study the pharmacokinetic and pharmacodynamic interactions between ML and mainstream hypoglycemic agents (e.g., metformin, SGLT2 inhibitors, insulin), and to establish a clear clinical guideline for the use of ML. More importantly, future research should focus on overcoming the bottleneck of industrialization by building a whole chain quality control system (including variety selection, process standardization, and multi-indicator fingerprinting quality control) from farm to clinic, and utilizing nano-delivery and other technological innovations in dosage forms. The ultimate goal is to transform ML into a modern precision nutritional intervention tool with clear mechanism, controllable quality and precise efficacy through multidisciplinary collaboration, so as to provide a scientific and reliable dietary strategy for diabetes prevention and control.

## 7 Conclusion

In conclusion, the synergistic effects of multiple functional components (e.g., alkaloids, flavonoids and polysaccharides) in ML through multi-targets and multi-pathways, such as inhibition of digestive enzymes, modulation of glucose uptake, enhancement of insulin sensitivity and protection of pancreatic islet β-cells, provide a solid scientific basis for its adjunctive blood glucose control. Available evidence supports its potential as a safe and effective dietary supplement or functional food ingredient. However, more in-depth studies on the standardization of active ingredients, high-quality clinical long-term validation and exploration of deep molecular mechanisms are still needed in the future to promote its transition from traditional experience to modern evidence-based applications.
